# Chemical Standardization and Anti-Proliferative Activity of *Ardisia elliptica* Fruit against the HCT116 Human Colon Cancer Cell Line

**DOI:** 10.3390/molecules25051023

**Published:** 2020-02-25

**Authors:** Suchanuch Ondee, Pongtip Sithisarn, Supachoke Mangmool, Piyanuch Rojsanga

**Affiliations:** 1 Department of Pharmaceutical Chemistry, Faculty of Pharmacy, Mahidol University, Bangkok 10400, Thailand; suchanuch@yahoo.com; 2 Department of Pharmacognosy, Faculty of Pharmacy, Mahidol University, Bangkok 10400, Thailand; pongtip.sithj@mahidol.ac.th; 3 Department of Pharmacology, Faculty of Science, Mahidol University, Bangkok 10400, Thailand; supachoke.man@mahidol.ac.th

**Keywords:** standardization, *Ardisia elliptica*, anti-proliferative, embelin, colon cancer, HCT-116, XIAP

## Abstract

The present study is intended to carry out the chemical standardization and evaluation of the anti-proliferative activity of *A. elliptica* fruit extract. *A. elliptica* fruit powder was extracted with ethanol. The obtained extract was assessed for total phenolic content using the Folin–Ciocalteu method. Moreover, a simple, accurate, and precise reversed phase high-performance liquid chromatographic method was developed and validated to determine the embelin content of *A. elliptica* fruit extract. Then, the extract and embelin were investigated for their anti-proliferative effect against HCT-116 cells. Finally, the mechanisms of inhibition of the extract and embelin on the mRNA expression of pro-apoptotic genes Bad, Bax, and Caspase-8 and anti-apoptotic genes c-IAP1, Mcl-1, and XIAP were determined by real-time qRT-PCR. The phenolic content and embelin content of the extract were 5.20 ± 0.01 g of gallic acid equivalent per 100 g of dried fruit (g% GAE) and 5.57 ± 0.56 mg/g of extract, respectively. The extract and embelin showed strong anti-proliferative effects on HCT-116 cells with 50% inhibition concentration (IC_50_) values of 19.16 ± 1.09 µg/mL and 25.93 ± 1.75 µg/mL, respectively. The *A. elliptica* extract exhibited a significant increase in the mRNA level of Bad, Bax, and Caspase-8 and a significant decrease in c-IAP1, Mcl-1, and XIAP. Embelin showed a significant decrease in Mcl-1 and XIAP.

## 1. Introduction

Colon cancer, also known as bowel cancer and colorectal cancer, is uncontrolled cell growth in the colon or rectum [1]. Colon cancer is the fourth most common cause of death in industrialized countries and the third most common cancer around the world [2]. Even though there are enhanced diagnostic and therapeutic methodologies available, the morbidity and mortality of colon cancer is still substantial [3]. The limits of cancer screening and a poor prognosis in the advanced stages of colon cancer have caused a great deal of interest in exploring antitumor agents. Due to the reduced toxicity and cost effectiveness, herbal medicine has gradually attracted more consideration as an alternative cancer therapy [4]. There are many phytochemicals that have been identified for their modulating effects on these molecular targets including genistin, resveratrol, allicin, lycopene, capsaicin, curcumin, 6-gingerol, ellagic acid, catechins, cruciferous, ursolic acid, and eugenol [5,6,7,8].

*Ardisia elliptica* Thunb, known in Thailand as Ram Yai or Pi-lung-ga-sa, is a Thai medicinal plant that belongs to the Myrsinaceae family. It is a small-branched shrub tree with smooth and leathery texture leaves and pale violet flowers. Fruits are round, berry-like drupes that turn from red and dark purple to black when they are ripe. The fruits are edible and taste slightly astringent. *A. elliptica* is commonly found in Sri Lanka, China, Taiwan, and Southeast Asian countries especially: Thailand, Vietnam, Malaysia, Indonesia, and the Philippines [9]. *A. elliptica* is traditionally used for alleviating chest pains, the treatment of fever, diarrhea, liver poisoning, and parturition complications. The leaves and roots of this plant have traditionally been used in Southeast Asian herbal remedies. The decoction of the leaves of *A. elliptica* is used for treatment of pain in the region of the heart or to alleviate chest pains [10]. *A. elliptica* has more potency than aspirin in the inhibition of collagen-induced platelet aggregation by β-xamyrin contained in *A. elliptica* leaves [11]. The ethanolic extract of *A. elliptica* fruit exhibited anti-proliferative activity on SKBR3 human breast adenocarcinoma cell lines [12] and showed antioxidant and antidiarrheal activities [13]. There is also a report that highlighted the anticancer potential against liver cancer cells of tea extracts from the leaves of six species of *Ardisia* species of which *A. compressa*, *A. crenata*, and *A. japonica* promoted a high potential among the tested samples, but with an unclear mechanism of action [14]. There is also a study demonstrating the in vitro antibacterial and antioxidant effects of methanol extracts from the leaves and the fruits of *A. elliptica*. This report also suggested the presence of phenolics and flavonoids in these extracts of *A. elliptica* [15]. The phytochemical compounds contained in leaves of *A. elliptica* are bauerenol, α-amyrin, β-amyrin, and bergenin [16,17]. Syringic acid, isorhamnetin, β-amyrin, quercetin, and anthocyanin have been isolated from the fruit [18]. *A. elliptica* fruits contain a quinone derivative, embelin, as a major constituent. Myricetin, quercetin, norbergenin, kaempferol, quercetin 3-0-β-d-glucopyranoside, and gallic acid were also reported [19]. The safety of the extract of *A. elliptica* fruit was evaluated in an animal model. The oral administration of ethanolic extract of *A. elliptica* fruits at the dose of 5 g/kg promoted no acute toxicity in mice, while the subchronic toxicity study in Wistar rats, at doses of 20–2000 mg/kg/day, also did not promote any toxicity [20]. Even though *A. elliptica* fruit has many potential activities such as antimicrobial, antioxidant, and anti-proliferative activities, the effects of phytochemicals contained in *A. elliptica* fruit extract against colon cancer cells have not been fully defined. The present study aimed to determine the total phenolic and embeline contents as chemical parameters for the standardization of *A. elliptica* fruit extract, and to investigate the effects of the extract as well as embelin on the inhibition of cell proliferation in HCT-116 cells.

## 2. Results

### 2.1. Determination of Total Phenolic Contents in A. elliptica Fruit Extracts by Folin-Ciocalteu Method

The total phenolic content was calculated from a standard calibration curve of standard gallic acid and expressed as miligram of gallic acid equivalent per 1 g of dried extract (mg GAE/g). The total phenolic contents of the *A. elliptica* fruit were 52.0 ± 0.1 mg GAE/g dried extract.

### 2.2. Phytochemical Analysis of A. elliptica Fruit Extract by Thin Layer Chromagography (TLC)

**Figure 1:**
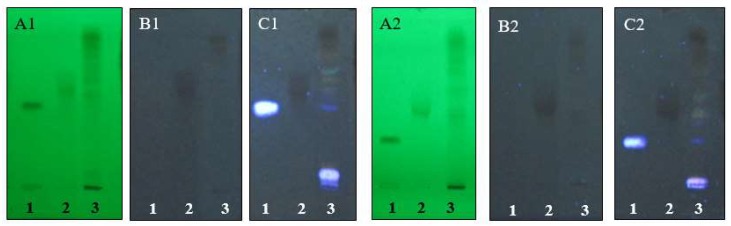
Thin layer chromagography (TLC) chromatogram of Ardisia elliptica fruit extract; 1 = gallic acid, 2 = embelin, 3 = Ardisia elliptica fruit extract, adsorbent: silica gel GF254. Solvent system: 1 = acetate:glacial acetic acid:formic acid:hexane (15:2:2:10, *v*/*v*/*v*/*v*), 2 = ethyl acetate:toluene:formic acid (9:10:2, *v*/*v*/*v*). detection: A = UV 254 nm, B = UV 366 nm, and C = NP/PEG under UV 366 nm. Band identification system 1: gallic acid (Rf = 0.46), embelin (Rf = 0.62). Band identification system 2: gallic acid (Rf = 0.26), embelin (Rf = 0.46).

*A. elliptica* fruit extract was phytochemically analyzed using two different solvent systems. The extract exhibited thin layer chromagography (TLC) fingerprints with the presence of chromatographic bands that corresponded to some phenolics and flavonoids, as shown in Figure 1. There were chromatographic bands that corresponded to gallic acid and embelin at retardation factor (Rf) values of 0.46 and 0.62 in solvent system 1 and 0.26 and 0.46 in solvent system 2, respectively. Moreover, there were chromatographic bands at Rf values of 0.68 and 0.52 in solvent system 1 and 2, respectively that appeared as yellow fluorescence bands after spraying with natural product/polyethylene glycol (NP/PEG) spray reagent and detected under UV 366 nm and chromatographic bands at Rf values of 0.63 and 0.42 in solvent system 1 and 2, respectively that appeared as orange fluorescence bands after spraying with NP/PEG spray reagent and detected under UV 366 nm. The presences of these chromatographic bands suggested the presences of flavonoids.

### 2.3. Phytochemical Analysis of A. elliptica Fruit Extract by Liquid Chromatography-Mass Spectrometry (LC-MS)

The peaks of gallic acid, quercetin, kaempferol, and embelin in *A. elliptica* fruit extract were identified by LC-MS technique. It was found that the LC-MS peak at the retention time of 0.77 min with molecular mass (negative mode) of 168.95 *m*/*z* and fragment ion of 124.95 and 79.1 *m*/*z* corresponded to gallic acid. Quercetin promoted the LC-MS peak at the retention time of 1.09 min with a molecular mass of 301.05 *m*/*z* and fragment ion of 106.9 and 151.15 *m*/*z*. Kaempferol promoted the LC-MS peak at the retention time of 1.40 min with a molecular mass of 285.05 *m*/*z* and fragment ion of 93 and 116.95 *m*/*z*, while embelin promoted the LC-MS peak at the retention time of 3.48 min with a molecular mass of 293.1 *m*/*z* and a fragment ion of 96.1 and 124 *m*/*z*. The results from LC-MS analysis corresponded to the results from TLC analysis suggesting that *A. elliptica* fruit extract contained gallic acid, embelin, and flavonoids, which could be quercetin and kaempferol.

### 2.4. Quantitative Analysis of Embelin in A. elliptica Fruit Extracts by High-Performance Liquid Chromatography (HPLC)

**Figure 2:**
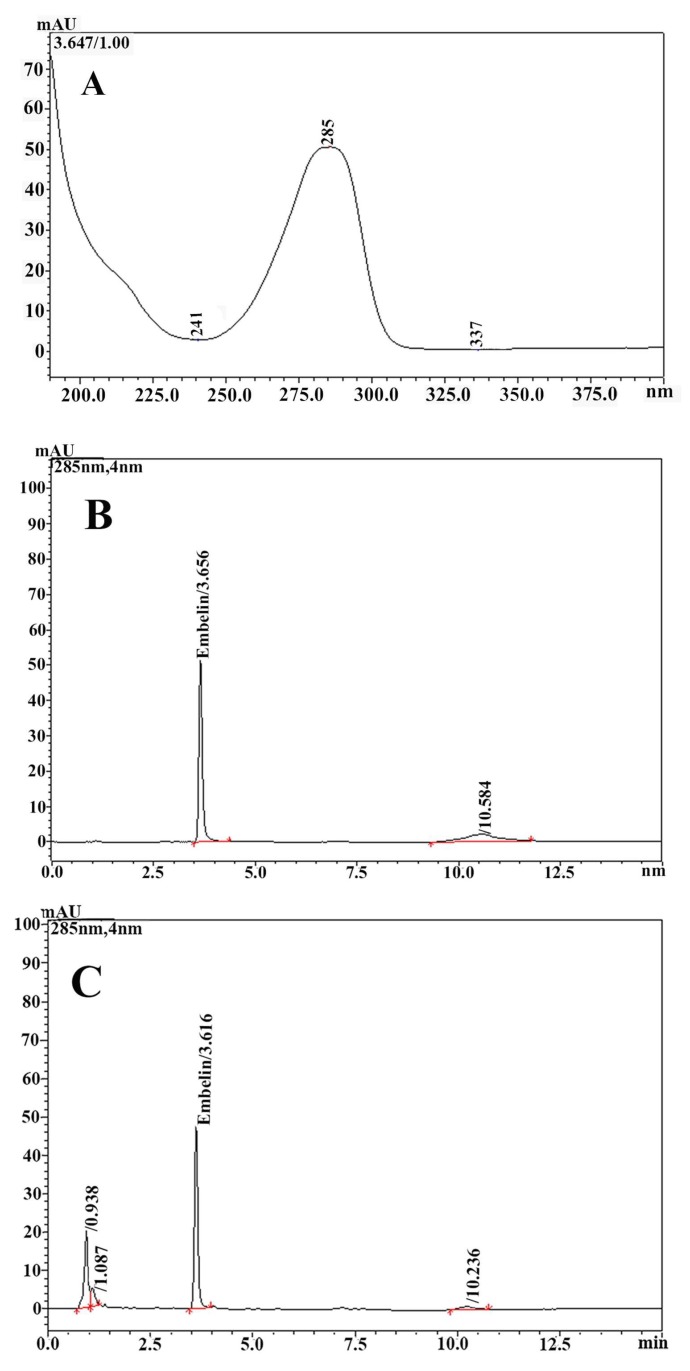
UV spectrum at 200–400 nm of standard embelin (**A**), HPLC chromatograms determined at 285 nm of standard embelin at a concentration of 20 µg/mL (RT = 3.66 min) (**B**), and HPLC chromatogram determined at 285 nm of *A. elliptica* fruit extract at a concentration of 1 mg/mL (retention time (RT) = 3.62 min) (**C**).

The HPLC chromatogram of the *A. elliptica* fruit extract exhibited a good separation of embelin from the other peak in the extract (Figure 2). The UV spectrum at 200–400 nm of the peak in the extract showed the retention matching the peak of the embelin standard with a retention time (RT) of 3.66 min, as shown in Figure 1. The calibration curve was linear in the range of 10–100 µg/mL. The linear equation was y = 17032x − 28094. The correlation coefficient (r) of the equation was 0.9989. The recovery of embelin in *A. elliptica* extract was performed on the sample solution spiked with three concentration levels of standard embelin at 10, 20, and 40 µg/mL. The ranges of recovery were 97.55–101.56% with a mean recovery of 99.48%. The repeatability and intermediate precision were determined from embelin content in the sample solution at a concentration of 1 mg/mL on the same day (*n* = 6) and three different days (*n* = 18), respectively. For repeatability, the % relative standard deviation (RSD) of embelin ranged from 0.45 to 0.80. The % RSD of the intermediate precision of embelin was 2.81. The limit of detection (LOD) of embelin was 0.25 µg/mL with the RSD of 0.01%. The LOQ of embelin was 1 µg/mL with the RSD of 0.01%. The sample solutions were kept at 15 °C in an autosample at 25 °C for 7 days. The embelin contents in the sample solutions were evaluated on days 1 and 7. The % RSD of embelin content in the extracts kept at 15 °C and 25 °C were 3.75 and 5.24, respectively. The sample solution of the *A. elliptica* fruit extract was determined to be stable in embelin for at least 7 days at 15 °C. In summary, the validated HPLC method was deemed suitable for quantifying embelin in the *A. elliptica* fruit extract.

The % yield of the extract was 22.3% *w*/*w* of the dried plant and the embelin content in the *A. elliptica* fruit was 5.57 ± 0.56 mg/g of dried extract.

### 2.5. Determination of Anti-Proliferative Activity of A. elliptica Fruit Extract

**Figure 3:**
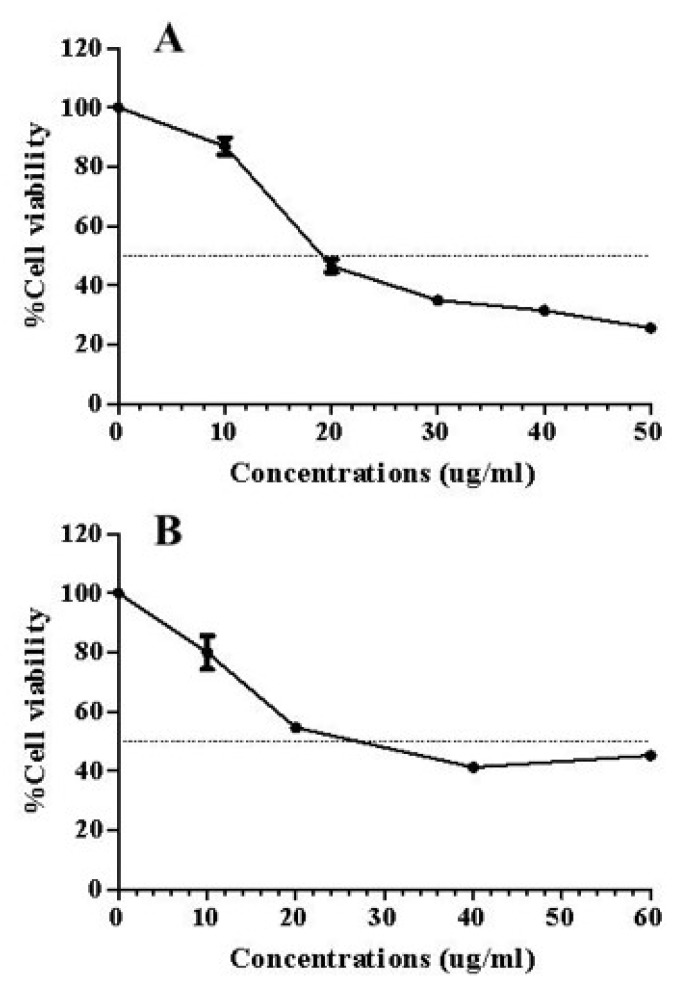
The anti-proliferative effects of *A. elliptica* fruit extract (**A**) and embelin (**B**) against HCT-116 cells after 48 h of incubation.

The anti-proliferative effect against HCT-116 cells treated with *A. elliptica* fruit extract and embelin is presented in Figure 3. As shown in Table 1, *A. elliptica* fruit extract had a lower 50% inhibition concentration (IC_50_) value compared to embelin, indicating that the extract possesses higher in vitro potency than embelin in inhibiting the proliferation of HCT-116 cells. When *A. elliptica* fruit extract and embelin were tested on normal cells (Vero cell), neither of them caused cytotoxicity at concentrations higher than three times the IC_50_ values for HCT-116 cells (Table 1).

### 2.6. Effects of A. elliptica Fruit Extract and Embelin on mRNA Expressions of Pro-Apoptotic and Anti-Apoptotic Genes

**Figure 4:**
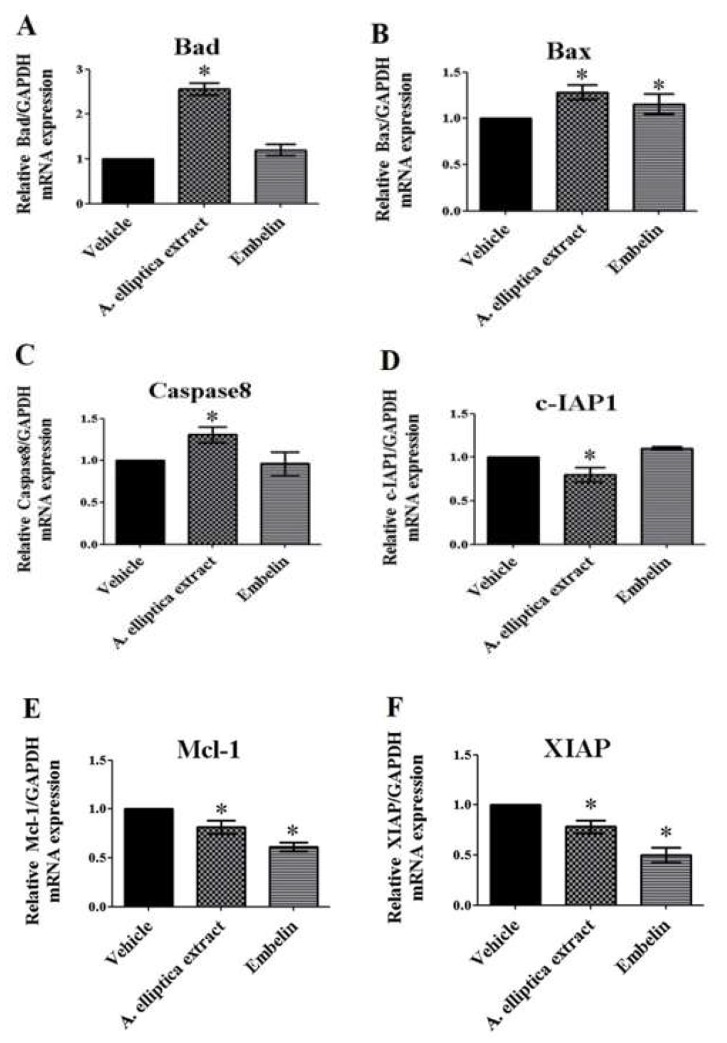
Effects of *A. elliptica* fruit extract and embelin on mRNA expressions of pro- and anti-apoptotic genes (**A**–**F**). Cells were treated with the vehicle (control), 10 µg/mL fruit extracts, or 20 µg/mL embelin for 12 h at 37 °C. After treatment, the total RNA was extracted from the cells, and the mRNA expression was analyzed using specific primers. The relative Bad (**A**), Bax (**B**), Caspase-8 (**C**), c-IAP1 (**D**), Mcl-1 (**E**), and XIAP (**F**) mRNA levels were quantified and shown as the mean ± SEM (*N* = 3). * *p* < 0.05 versus vehicle.

We next examined whether the treatment of HCT-116 cells with *A. elliptica* fruit extract and embelin is able to induce an apoptosis pathway. To achieve this hypothesis, HCT-116 cells were treated with either *A. elliptica* fruit extract (10 µg/mL) or embelin (20 µg/mL) for 12 h, and the mRNA expression of various pro-apoptotic and anti-apoptotic genes were measured. As shown in Figure 3, treatment with *A. elliptica* fruit extract significantly elevated the mRNA levels of Bad, Bax, and caspase-8, which are pro-apoptotic genes. In addition, HCT-116 cells treated with *A. elliptica* fruit extract resulted in a reduction in the mRNA expressions of c-IAP1, Mcl-1, and XIAP, which are anti-apoptotic genes. These results suggest that the extract from *A. elliptica* fruit induces an apoptosis pathway in HCT-116 cells. We also investigated the effects of embelin on mRNA expressions of several genes involved in apoptosis. The mRNA levels of Mcl-1 and XIAP were significantly reduced in embelin-treated HCT-116 cells (Figure 4), indicating that embelin acts as an inhibitor of the anti-apoptotic pathway.

## 3. Discussion

Natural products have been a significant source of novel compounds for the treatment of numerous diseases, with currently more than 60% of anticancer drugs having been developed based on the traditional knowledge of ancient cultures [21,22]. Since the incidence and death rate of colon cancer is high, it is important to prevent cancer by reducing carcinogenesis using cancer chemopreventive drugs in terms of public health and economics. Thus, it is essential to evaluate natural products systemically for the identification of molecular targets as a first step in finding active compounds and consequently exploring their mechanism of action.

In our previous studies, the anti-proliferative activity on colorectal cancer HCT-116 cells of decoction and 95% ethanol extracts from 30 Thai edible plants collected from Sa Keao province, Thailand using a Cell Titer 96® Aqueous One Solution Cell Proliferation Assay were determined. It was found that the ethanolic extract of *A. elliptica* fruits showed the highest activity with an IC50 value less than 20 µg/mL. The results suggest that *A. elliptica* fruits possessed anti-proliferative activity, which could be useful for further application as cancer chemopreventive and cancer therapeutic agents. However, there is still no report about the mechanism of action related to the anti-proliferative effect of *A. elliptica* fruit extract.

In the present study, we continued our experiments to search for the underlying mechanisms of the anti-proliferative effect of *A. elliptica* fruit extract on HCT-116 cells. The extract was rich in total phenolic, and embelin was found to be the major compound in *A. elliptica* fruit extract analyzing by the proposed HPLC method. Interestingly, the *A. elliptica* fruit extract exhibited a stronger inhibitory effect than that of embelin, which suggested that there may be other compounds responsible for the anti-proliferative activity of the extract. Phytochemical analysis by both TLC and LC-MS techniques revealed that there were some other phenolics and flavonoids in *A. elliptica* fruit extract such as gallic acid, quercetin, and keaempferol. These compounds could also play important roles in inhibitory effects on cancer cell lines. Some reports showed that gallic acid induces apoptosis in A375.S2 human melanoma cells through caspase-dependent and caspase-independent pathways, and quercetin induces apoptosis in a caspase-3-dependent pathway in leukemia cell line (HL-60), while kaempferol was found to bind with caspases through an allosteric mode of competitive inhibition using both in silico computer docking technique and in vitro assay [23,24,25]. Moreover, many studies have shown that the overall effect of plant extracts can be cause by mixtures of compounds with synergistic or additive effects [26,27,28].

To evaluate the mechanism of the anti-proliferative effect, real-time qRT-PCR was used to determine the mRNA expressions of the pro-apoptotic genes Bad, Bax, and Caspase-8 and the anti-apoptotic genes c-IAP1, Mcl-1, and XIAP. *A. elliptica* fruit extract significantly increased the mRNA levels of the pro-apoptotic genes—Bad, Bax, and Caspase-8—but significantly decreased the mRNA levels of the anti-apoptotic genes c-IAP1, Mcl-1 and XIAP. Meanwhile, embelin showed a significant decrease in the mRNA expression of only Mcl-1 and XIAP. Embelin is an inhibitor of XIAP, and it is found in *A. elliptica* fruit extract. It has been shown to exhibit anti-proliferative and anti-cancer effects on various cancer cells [29,30]. The previous study demonstrated that treatment with embelin, a quinine derivative isolated from the *Embelia ribes* plant, caused the inhibition of cell proliferation and the induction of apoptosis in the leukemic cell lines K562 and U937 [31]. This previous study concluded that embelin mediated the inhibition of cell proliferation due to the induction of apoptosis. Consistent with this previous study, our study demonstrated that *A. elliptica* fruit extract containing embelin exhibited both anti-proliferative effects and apoptotic effects by enhancing the production of pro-apoptotic Bad and Bax and suppressing the synthesis of anti-apoptotic c-IAP1, Mcl-1, and XIAP, as determined by the measurement of mRNA levels. Our study also showed that treatment with *A. elliptica* fruit extract significantly induces caspase-8 activity, while embelin treatment tends to increase caspase-8 activity in HCT-116 cells. In the same way, treatment with embelin activates the activation of caspase-9 and the sequential activation of caspase-3 in leukemic cell lines [28]. Thus, embelin is one of potential active compounds found in *A. elliptica* fruit extract that induces apoptosis through the caspase signaling pathway.

Several previous studies have been demonstrated that XIAP is a potential therapeutic target for various types of cancer, as an overexpression of XIAP is found in many cancer cells [31,32]. Embelin acts as an inhibitor of XIAP in a variety of cancers. Both previous studies and our present study have demonstrated that embelin mediates apoptosis and induces cell death in cancer cells [31,32]. Moreover, our study showed that *A. elliptica* fruit extract has similar anti-proliferative effect compared to that of embelin in HCT-116 cells without cytotoxicity to normal cells (Vero cell). The results suggested that the elevated inhibitory effect of *A. elliptica* fruit extract may be a result of the combined action of other constituents and embelin that work synergistically with different pathways. However, the apoptotic effects of *A. elliptica* extract and embelin came from the results of an mRNA analysis. Therefore, a protein analysis using a Western blot assay is necessary for investigating the protein levels of pro-apoptotic and anti-apoptotic genes following treatment with this extract.

## 4. Materials and Methods

### 4.1. Chemicals and Reagents

Ethanol (95%, AR grade), acetonitrile (HPLC grade), and methanol (HPLC grade) were purchased from RCI Labscan, Bangkok, Thailand. Methanol (AR grade) was purchased from Honeywell, Charlotte, NC, USA. Deionized water was obtained using a water purification system from Thermo Scientific Co. (Waltham, MA, USA). Gallic acid, quercetin, and kaempferol were purchased from the TOKYO chemical industry Co., Ltd., Tokyo, Japan. Sodium carbonate, embelin, and doxorubicin hydrochloride were purchased from Sigma-Aldrich, St. Louis, MO, USA. Folin–Ciocalteu, orthophosphoric acid, and dimethylsulfoxide were obtained from Merck, Darmstadt, Germany. McCoy’s 5A medium, Eagle’s Minimum Essential Medium, and Dulbecco’s phosphate-buffered saline were purchased from Lonza, Walkersville, MD, USA. Penicillin, streptomycin, Dulbecco’s Modified Eagle Medium, fetal bovine serum, and 0.25% Trypsin-EDTA (Ethylenediaminetetraacetic acid) were purchased from Gibco, Waltham, MA, USA.

### 4.2. Plant Material and Extract Preparations 

The fruits of *A. elliptica* were collected from the Siri Ruckhachati Nature Park (latitude: 13°79′ N, longitude: 100°32′ E, height above mean sea level: 16 feet), Salaya Campus, Mahidol University, Buddhamonthon district, Nakhonpathom Province, Thailand in November 2014. The samples were washed and dried in a hot air oven (Memmert, Eagle, WI, USA) at 60 °C for 6–8 h. The dried samples were ground into powder and passed through a sieve with mesh number 20 (sieve size of 0.85 mm). Three hundred grams of *A. elliptica* fruit powder was macerated with 95% ethanol using an electric shaker at room temperature for 6 h, with a solid-to-liquid ratio of 1:5 *w*/*v*. The extract solution was filtered through a Whatman filter paper No. 1 and evaporated to dryness on a water bath (Memmert, Eagle, WI, USA) at 60 °C.

### 4.3. Determination of Total Phenolic Contents in A. elliptica Fruit Extracts Using Folin–Ciocalteu Method

Ten milligrams of gallic acid were weighed in a 10-mL volumetric flask and adjusted with 20% methanol (the stock gallic acid solution was 1 mg/mL). The stock standard solution was diluted to a concentration of 0.1 mg/mL. The working standard solutions at concentrations of 1–10 µg/mL were prepared for the calibration curve. The solution of *A. elliptica* fruit extracts was prepared in 50% ethanol at a concentration of 500 μg/mL. Two milliliters of gallic acid or sample solutions were transferred to 10-mL volumetric flasks, and 4 mL of 0.2 N Folin–Ciocalteu solution was added. After 5 min, 3.2 mL of 7.5% *w*/*v* sodium carbonate solution was added. The mixtures were adjusted to volume with distilled water and incubated at room temperature for 90 min. The absorbance of each solution was measured using a UV-Vis spectrophotometer (Shimadzu, Tokyo, Japan) at a wavelength of 760 nm. The total phenolic contents were calculated from the standard calibration curve of gallic acid and expressed as grams of gallic acid equivalent per 100 g of dried fruit (g% GAE). The experiment was performed in triplicate and expressed as mean ± standard deviation (SD).

### 4.4. Phytochemical Analysis of A. elliptica Fruit Extracts by Thin Layer Chromagography (TLC)

*A. elliptica* fruit extract was spotted on a precoated silica gel 60 GF254 TLC plates. The plates were developed in two solvent systems, which were ethyl acetate:glacial acetic acid:formic acid:hexane (15:2:2:10, *v*/*v*/*v*/*v*) and ethyl acetate:toluene:formic acid (9:10:2, *v*/*v*/*v*). The developing distance was 65 mm. After being removed from the developing chamber, the TLC plates were air dried in a fume hood and examined under UV light at wavelengths of 254 and 366 nm and under UV light at a wavelength of 366 nm after spraying with a natural product/polyethylene glycol (NP/PEG) reagent. The TLC fingerprints of *A. elliptica* fruit extract were recorded.

### 4.5. Phytochemical Analysis of A. elliptica Fruit Extracts by Liquid Chromatography Tandem Mass Spectrometry

LC-MS-MS analysis of extract from *A. elliptica* fruits was conducted using a Shimadzu LCMS-8030 triple quadrupole mass spectrometer equipped with electrospray ionization (Shimadzu, Tokyo, Japan). An InertSustain C8 analytical column (2.10 mm i.d. × 100mm, 3 µm) was used. Isocratic elution was performed with 0.1% formic acid in water (solvent A) and acetonitrile (solvent B) (1:1) at a constant flow rate of 0.3 mL/min. The column temperature was 30 °C with an injection volume of 1 µL.

### 4.6. Quantitative Analysis of Embelin in A. elliptica Fruit Extracts by High-Performance Liquid Chromatography (HPLC)

#### 4.6.1. Instrumentation

The high-performance liquid chromatography (HPLC) analysis was performed using a HPLC (Shimadzu, Tokyo, Japan) instrument with two LC-20ADXR pumps, a CMB-20A system controller, a CTO-20A column oven, a SIL-20AC auto-sampler, a DUG-20A degassing unit, and a SPD-M20A diode array detector.

The quantitative analysis of embelin was performed using the X-Terra® C18 column (3.9 × 150 mm, 5 μm). A mobile phase comprising of 0.1% (*v*/*v*) orthophosphoric acid in water (Solvent A) and acetonitrile (Solvent B) was used. The gradient programs were linear from 70% to 90% Solvent B for 6 min, remaining at 90% Solvent B for 1 min, and then from 90% to 70% for 3 min, followed by the equilibration of the column at 70% Solvent B for 5 min. The conditions used for analysis were a flow rate of 1.2 mL/min, an injection volume of 10 μL, and a running time of 15 min. The temperature of the column oven was set at 40 °C and the auto-sampler was set at 15 °C. The detection wavelength was 285 nm with a photodiode array detector (PDA). UV-Vis absorption spectra were recorded on-line from 200 to 400 nm during the HPLC analysis. The data were processed using Shimadzu’s Lab Solutions software.

#### 4.6.2. Method Validation

The HPLC method for the quantitative determination of embelin in *A. elliptica* fruit extracts was validated in term of linearity, accuracy, precision, limit of detection (LOD), and limit of quantitation (LOQ) according to the Association of Official Analytical Chemists’ (AOAC) guidelines for the single laboratory validation of chemical methods for dietary supplements and botanicals [33].

For the standard preparation, the primary stock solution of standard embelin was prepared with a concentration of 1 mg/mL in methanol. The secondary stock solution was prepared with a concentration of 100 µg/mL by transferring 2.5 mL of the primary stock solution into a 25-mL volumetric flask and adjusting to volume with methanol. The working standard solutions were prepared by diluting the secondary solution to a concentration of 10 to 80 µg/mL.

For the sample preparation, the *A. elliptica* fruit extract was weighed and dissolved in methanol to obtain the primary sample solution at a concentration of 10 mg/mL. The working sample solutions were prepared by diluting the primary solution to a concentration of 1 mg/mL.

For selectivity, the HPLC chromatograms of the standard solution and the sample solution were separately analyzed. The UV spectra at 200–400 nm of the peak at the retention time corresponding to embelin in the sample were compared with the UV spectra of the standard peak.

The linearity of the method was evaluated by analyzing the series of standard embelin in methanol. Three calibration curves, including six concentration levels, were in the range of 10–100 µg/mL. The standard curves were obtained by plotting the concentrations (x-axis) versus the peak areas of each concentration (y-axis). The slope and intercept values and the correlation coefficient (r) were calculated using the least-square linear regression method (*r* ≤ 0.99).

The accuracy of the method was evaluated in terms of the recovery using three different concentrations of standard embelin (10, 20, and 40 µg/mL) spiked into the working sample solution (1 mg/mL) and adjusted with methanol. Each concentration was performed in triplicate. The percentage of recovery of each concentration of embelin was calculated.

Repeatability and intermediate precision were obtained by an evaluation of the content of embelin in *A. elliptica* fruit extract solution at a concentration of 1 mg/mL. The analysis was performed on the same day (*n* = 6) for repeatability and on three different days (*n* = 18) for intermediate precision. The results were expressed as a percentage of relative standard deviation (%RSD). The limit of detection (LOD) and limit of quantitation (LOQ) of the analyzed method were investigated based on the signal-to-noise ratio. LOD and LOQ were defined as the lowest concentration of the standard, which exhibited the ratios of signal to noise as 3:1 and 10:1, respectively. The stability of the sample solution was evaluated by comparing the content of embelin in the sample solution, which was kept at temperatures of 15 °C and 25 °C on Days 1 and 7.

The embelin content in *A. elliptica* fruit extract was determined by the validated HPLC method. The working sample solutions were prepared at a concentration of 1 mg/mL and filtered using a 0.2 μm polytetrafluoroethylene (PTFE) membrane filter. All sample solutions were analyzed using the HPLC conditions previously described. The content of the embelin was calculated from the linear equation using the standard curve of embelin solutions (at a concentration range of 10 to 100 µg/mL) and expressed as milligram per gram of dried extract.

### 4.7. Cell Culture and Cell Proliferation Assay

Human colorectal carcinoma (HCT-116) and African green monkey kidney (Vero) cells were purchased from the American Type Culture Collection (ATCC, Rockville, MD, USA). The HCT-116 cells were cultured in McCoy’s 5A medium supplemented with 10% fetal bovine serum (FBS) and 100 unit/mL of penicillin and 100 µg/mL of streptomycin. The Vero cells were cultured in the 25 cm^2^ tissue culture flask, which contained Eagle’s Minimum Essential Medium (EMEM) supplemented with 10% fetal bovine serum (FBS), 100 unit/mL of penicillin, and 100 µg/mL of streptomycin. The cells were cultured at 37 °C in a humidified 5% CO_2_ incubator (Shel Lab, Cornelius, OR, USA).

The anti-proliferative effects of embelin and *A. elliptica* fruit extract were investigated on the HCT-116 and Vero cells using a CellTiter 96® Aqueous One Solution Cell Proliferation Assay (Promega, Madison, WI, USA). The HCT-116/Vero cell suspensions at a concentration of 2 × 104 cells/mL were prepared in a medium supplemented with 10% FBS. One hundred microliters of HCT-116/Vero cells (2 × 104 cells/mL) were seeded into a 96-well plate (2 × 103 cells/well). After 24 h of incubation at 37 °C in 5% CO_2_ incubator, the cells were treated with 100 μL of the extract at various concentrations. The extract and embelin was separately weighed and dissolved in dimethylsulfoxide (DMSO) as the stock solution. Then, it was diluted to concentrations of 10–60 µg/mL using the medium supplemented with 10% FBS. Negative control groups were treated with 0.5% DMSO in the medium instead of the extract. Doxorubicin hydrochloride at a concentration of 3 μg/mL was used as a positive control. After incubation for 48 h, 20 µL of CellTiter 96® Aqueous One solution was added into each well, and the 96-well plate was incubated at 37 °C in 5% CO_2_ incubator for 1 h. The absorbance was determined using a microplate reader (PerkinElmer, Waltham, MA, USA) at the wavelength of 490 nm. The percentage (%) of inhibition was calculated according to the equation below:

(1) $$\%\;\mathrm{inhibition}=1-[\frac{\mathrm{Absorbance}\;\mathrm{of}\;\mathrm{sample}}{\mathrm{Absorbance}\;\mathrm{of}\;\mathrm{control}}]\times 100.$$

Each sample was assayed in six replicates. The 50% inhibition concentration (IC_50_ value) was calculated from the linear equation between the percentages of cell viability versus *A. elliptica* fruit extract concentration.

### 4.8. Determination of Pro-Apoptotic and Anti-Apoptotic mRNA Expressions Using Real-Time qRT-PCR Technique

The HCT-116 cells were treated with *A. elliptica* fruit extract (10 µg/mL) or embelin (20 µg/mL) solutions in a serum-free medium for the indicated time. After treatment, RNA extraction was performed by using the RNeasy Mini kit (Qiagen). Real-time quantitative reverse transcription PCR (real-time qRT-PCR) system was performed using the KAPA SYBR FAST One-step RT-qPCR kit (KAPA biosystems, Wilmington, DE, USA) and Mx3005p Real Time PCR system (Agilent Technology, Palo Alto, CA, USA). Relative mRNA levels were examined by the comparative cycle threshold (CT) method and normalized to the housekeeping gene glyceraldehyde-3-phosphate dehydrogenase (GAPDH). We designed and synthesized the primers of pro-apoptotic and anti-apoptotic genes as shown in Table 2.

### 4.9. Statistical Analysis

Data were expressed as mean ± SEM (standard error of mean). Statistical analysis was assessed using one way analysis of variance (ANOVA) followed by the Tukey multiple comparison test or unpaired Student *t*-test as appropriate, and the mean was considered significantly different when *p* < 0.05.

## 5. Conclusions

**Figure 5:**
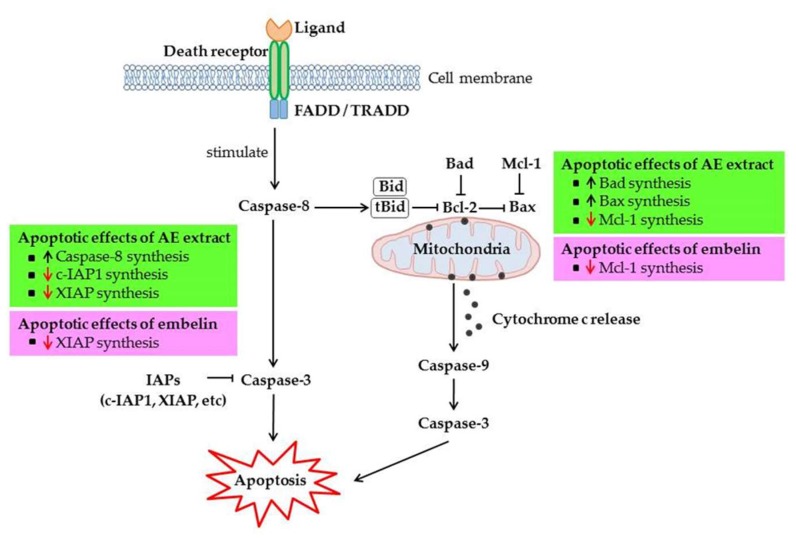
Schematic diagram illustrating the mechanisms of *A. elliptica* fruit extract (AE extract) and embelin on apoptotic pathway in HCT-116 cells.

Our study demonstrates the chemopreventive potential of *A. elliptica* fruit extract and embelin. As shown in Figure 5, the mechanism underlying *A. elliptica* fruit extract-induced anti-proliferative activity against HCT-116 cells could be the induction of apoptosis via an increase in the mRNA level of pro-apoptotic genes and a decrease in the mRNA level of anti-apoptotic genes. Embelin was found to be one of the active compounds and could be used as a chemical marker to quality control *A. elliptica* fruit extract.

## Figures and Tables

**Table 1:** The anti-proliferative effects of *A. elliptica* fruit extract, embelin, and doxorubicin toward HCT-116 and Vero cells as determined by a cell proliferation assay.

Samples	Cell Lines, IC_50_ Values in μg/mL	
	Vero	HCT-116
*A elliptica* fruit extract	>100	19.16 ± 1.09 ^a *^
Embelin	>100	25.93 ± 1.75 ^b^
Doxorubicin	<3	<3

* Different letters in the same column are significantly differences (*p* < 0.05, unpaired student *t* test).

**Table 2:** The specific primers for the human pro-apoptotic and anti-apoptotic genes.

Gene Specific Primer	Sequences	
Bad	Sense	5′-CCCAGAGTTTGAGCCGAGTC-3′ ^a, b, c, d^
	Antisense	5′-CCCATCCCTTCGTCGTCCT-3′
Bax	Sense	5′-CCCGAGAGGTCTTTTTCCGAG-3′
	Antisense	5′-CCAGCCCATGATGGTTCTGAT-3′
Caspase-8	Sense	5′-AGAGTCTGTGCCCAAATCAAC-3′
	Antisense	5′-GCTGCTTCTCTCTTTGCTGAA-3′
c-IAP1	Sense	5′-GAATACTCCCTGTGATTAATGGTGCCGTGG-3′
	Antisense	5′-TCTCTTGCTTGTAAAGACGTCTGTGTCTTC-3′
Mcl-1	Sense	5′-AAGCCAATGGGCAGGTCT-3′
	Antisense	5′-TGTCCAGTTTCCGAAGCAT-3′
XIAP	Sense	5′-GCACGAGCAGGGTTTCTTTATACTGGTG-3′
	Antisense	5′-CTTCTTCACAATACATGGCAGGGTTCCTC-3′
GAPDH	Sense	5′-CCCCTTCATTGACCTCAACT-3′
	Antisense	5′-TTGTCATGGATGACCTTGGC-3′

^a^ A: adenine, ^b^ C: cytosine, ^c^ G: guanine, ^d^ T: thymine.
